# The rising death burden of atrial fibrillation and flutter in low-income regions and younger populations

**DOI:** 10.3389/fepid.2023.1122790

**Published:** 2023-06-05

**Authors:** Ye-Mao Liu, Wenxin Wang, Xingyuan Zhang, Fang Lei, Juan-Juan Qin, Xuewei Huang, Ruyan Li, Lijin Lin, Mingming Chen, Yan-Xiao Ji, Peng Zhang, Xiao-Jing Zhang, Zhi-Gang She, Jingjing Cai, Chengsheng Xu, Zhengjun Shen, Hongliang Li

**Affiliations:** ^1^ Department of Cardiology, Huanggang Central Hospital, Huanggang, China; ^2^ Institute of Model Animal, Wuhan University, Wuhan, China; ^3^ Department of Cardiology, Renmin Hospital of Wuhan University, School of Basic Medical Science, Wuhan University, Wuhan, China; ^4^ Northfield Mount Hermon School, Gill, MA, United States; ^5^ Medical Science Research Center, Zhongnan Hospital of Wuhan University, Wuhan, China; ^6^ Department of Cardiology, The Third Xiangya Hospital, Central South University, Changsha, China

**Keywords:** atrial fibrillation and flutter, global burden of disease, metabolic risk factors, early death, prevention

## Abstract

**Objective:**

The aim of the study was to depict the global death burden of atrial fibrillation and/or flutter (AFF) between 1990 and 2019 and predict this burden in the next decade.

**Methods:**

We retrieved annual death data on cases and rates of AFF between 1990 and 2019 from the Global Burden of Disease (GBD) Study 2019 and projected the trends for 2020–2029 by developing the Bayesian age-period-cohort model.

**Results:**

The global number of deaths from AFF increased from 117,038.00 in 1990 to 315,336.80 in 2019. This number is projected to reach 404,593.40 by 2029. The age-standardized mortality rates (ASMRs) of AFF have increased significantly in low- to middle-sociodemographic index (SDI) regions, which will surpass that in high SDI regions and reach above 4.60 per 100,000 by 2029. Globally, women have a higher ASMR than men, which is largely attributed to disproportionately higher mortality in women than men in lower SDI regions. Notably, AFF-related premature mortality continues to worsen worldwide. A pandemic of high systolic blood pressure and high body mass index (BMI) largely contributes to AFF-associated death. In particular, low- to middle-SDI regions and younger populations are increasingly affected by the rapidly growing current and future risk of high BMI.

**Conclusion:**

The global death burden of AFF in low-income countries and younger generations have not been sufficiently controlled in the past and will continue growing in the future, which is largely attributed to metabolic risks, particularly for high BMI. There is an urgent need to implement effective measures to control AFF-related mortality.

## 1. Introduction

Atrial fibrillation and/or flutter (AFF) is the most common cardiac arrhythmia, which adds a mortality of 2% to cardiovascular disease (CVD)-related death (1). In past decades, given the aging population and the surge in AFF-associated risk factors, such as hypertension and obesity, the prevalence of AFF has risen considerably (2). Although the global rates of incidence and disability-adjusted life years have declined in a recent study (3), worryingly, AFF-related mortality is still growing rapidly based on our further investigation. Thus, the current death burden due to AFF merits particular attention. Our group has had a long interest in metabolic diseases and CVDs (4–10), thus the unfavorable situation of AFF inspired us to depict the past, current, and future scenery of the global burden of AFF, as well as the extent to which crucial risk factors contributed to the changes in AFF burden over time.

Notably, the younger generations have suffered from accumulated risk exposure to metabolic diseases in recent decades, which has largely contributed to the high prevalence of CVD in this population and an increased number of premature deaths (11). However, early death due to AFF has not been well documented thus far.

To fill these knowledge gaps, we analyzed the absolute number of deaths and age-standardized mortality rates (ASMRs) of AFF in 204 countries and territories between 1990 and 2019 using the results of the Global Burden of Disease (GBD) Study 2019 and predicted the trend in the death burden of AFF in the next decade by developing the Bayesian age-period-cohort (BAPC) model, thus to help enact effective strategies to prevent and intervene AFF-related death.

## 2. Method

### 2.1. Study data

The annual number of deaths and ASMRs of AFF by sex, age, region, and country between 1990 and 2019 were obtained online from the Global Health Data Exchange (GHDx) query tool (http://ghdx.healthdata.org/gbd-results-tool). The GBD 2019 database provides comprehensive and systematic estimations of 369 diseases and 87 attributable risk factors across 204 countries and territories over time (12, 13). According to the sociodemographic index (SDI), these countries and territories are categorized into five quintiles: low, low-middle, middle, high-middle, and high. The detailed country information of five SDI regions is presented in Supplementary Table S1. In addition, the GBD 2019 divides countries and territories into 21 GBD regions on the basis of their geographic locations. This study complied with the Guidelines for Accurate and Transparent Health Estimates Reporting (GATHER) statement, and the corresponding checklist is presented in Supplementary Table S2.

Data for further verifying the trend in early death were obtained from the World Health Organization (WHO) mortality database (https://www.who.int/data/data-collection-tools/who-mortality-database), which provides age- and sex-specific aggregated mortality data transmitted by national authorities for each underlying cause of death (14). The latest released files containing the detailed mortality data for the 10th revision of the International Classification of Diseases (ICD-10) were chosen to be analyzed.

Ethics approval and informed consent were not required for this study because of public accessibility to the data.

### 2.2. Definitions

The detailed diagnosis and confirmation methods of the GBD 2019 are available online (https://doi.org/10.1016/j.jacc.2020.11.010). AFF was determined according to standard case definitions and electrocardiography findings (1). Death data were obtained from the national vital registration database, verbal autopsies, or household mortality survey records. Each death is determined to the most detailed cause of death and coded by International Classification of Disease (ICD) detail coding systems: ICD-8, ICD-9, or ICD-10. Death due to AFF included causes coded as I48-I48.92 (ICD-10) and 427.3 (ICD-9) in GBD 2019 (https://ghdx.healthdata.org/record/ihme-data/gbd-2019-cause-icd-code-mappings).

Death between the ages of 30 and 70 years is the UN-recommended metric for representing premature death (15). However, because the age grouping interval for the GBD is 5 years (30–34, 35–39, … 65–69, 70–74, …), in reality, we defined premature death as death between the ages of 30 and 69 years.

### 2.3. GBD estimation methods

The conceptual and analytical framework for GBD, hierarchy of death causes and risk factors, and detailed estimation methods are available elsewhere (1, 12, 13). In brief, statistical methods, including misclassification correction, garbage code redistribution, and noise reduction algorithms, were adopted to reduce heterogeneity and improve the comparability of the data (16). The cause-specific mortality rate was estimated using the Cause of Death Ensemble model (CODEm), a highly integrated tool that runs many rounds of models on the same input data, and the ensemble of models that best reflects the available input data is finally chosen (17). The 95% uncertainty intervals (UIs) were calculated to reflect both random and systematic errors in statistical modeling.

The GBD study applied the comparative risk assessment framework (CRA) to quantify death burdens attributable to four levels of 87 risk factors (13). In brief, a causal relationship and the relative risk value between the risk-outcome pair were first determined according to sufficient evidence. Likewise, based on the exposure data of each risk factor disclosed in the population-based survey or report, a Bayesian meta-regression model (DisMod-MR 2.1) and spatiotemporal Gaussian process regression model were applied to estimate the mean exposure level of the risk. This process also included the determination of the theoretical minimum risk exposure level. Then, the population-attributable fraction was estimated according to population-specific parameters, including age, sex, region, and year, included in the input data. Finally, the abovementioned indices all served as input data into a specific model that estimated the disease burden attributable to a certain risk factor. This entire process was accompanied by various corrections of potential deviations.

### 2.4. Statistical analysis

#### 2.4.1. Forecasting model development and validation

We first collected the number of deaths for all ages (in 5-year intervals) at the global, regional, and national levels between 1990 and 2019 and restored the corresponding annual total populations. The GBD World Population Age Standard Population was later used to standardize the death rate of AFF.

Age-period-cohort (APC) models are increasingly frequently used in disease projection (18). Assuming that there is a multiplicative effect of age, period, and cohort, the trend in the age-adjusted death rate was modeled using the APC method:

$$Y_{\mathrm{at}}∼B(n_{\mathrm{at}},p_{\mathrm{at}})$$

$$n_{\mathrm{at}}=\log\left(\frac{\;p_{\mathrm{at}}}{1-p_{\mathrm{at}}}\right)=\mu +\theta_{a}+\phi_{t}+\varphi_{k}$$

The counts of deaths *Y*_at_ in age group *a* during time period *t* are assumed to follow a binomial distribution with parameters *p*_at_ and *n*_at_. The parameter *n*_at_ is the known population size of age group *a* at time period *t,* and *p*_at_ is the unknown mortality probability. Since the death rate is given per year while the age groups are depicted in 5-year intervals, *k *= *k* (*a*, *t*) = 5 × (*A* − *a*) + *t*, in this study, *A* = 14 (30–95+ years with an interval of 5 years), and *P* = 30 (1990–2019). The logit of the mortality probability is decomposed into an intercept *µ*, age effect *θ_a_*, period effect *ϕ_t_*, and cohort effect *φ_k_* (19). However, covariance among the three effects leads to the problem of unidentifiability in the classical age-period-cohort model. Bayesian inference treats all unknown parameters as random with appropriate prior distributions. The BAPC model avoids the problem of unidentifiability in the classical APC model by including random effects. In addition, the superior predictive performance of the BAPC model compared to the Joinpoint model, Poisson regression, and other prediction models has been previously verified (20, 21). In our study, we conducted the BAPC analysis with integrated nested Laplace approximation (INLA) (“BAPC” and “INLA” in R packages). The Gaussian second-order random walk (RW2) was used to adjust for overdispersion by assuming inverse-gamma prior distribution of our data, including age, period, and cohort effects (modeled by an RW2) (a detailed description about RW2 is given in Supplementary Materials).

The absolute percentage deviation (APD) was used to measure predictive deviance (21). We used the data between 1990 and 2013 to predict the trend in the death rate in 2014–2019 and compared it with the true values in the same period. The deviance was calculated as (*E* − *e*)/*e *× 100, where *E* and *e* denote the predicted and true values, respectively. The average APD values globally and in the five SDI regions and 21 GBD regions in the BAPC models are presented in Supplementary Table S3.

Some countries or territories have very small total populations, which often resulted in significant fluctuations in the mortality rate. To ensure smoothness in the forecast, those countries or territories were excluded, and a total of 167 countries or territories were finally included.

#### 2.4.2. Verification of the trends in AFF-related premature death using the WHO mortality database

We collected data on the number of deaths and the total population from the WHO mortality database. For countries with available AFF-related death data, those who had the year of the total population mismatching the year of the number of deaths, incoherent reports, and a time span shorter than 8 years were excluded. Finally, death data from Africa, South America, and Antarctica were lacking, and several countries with better data quality and larger populations from the remaining continents were selected to present the trends in the premature death rate: the United States, the United Kingdom, Italy, Denmark, the Czech Republic, Thailand, Uzbekistan, Egypt, Australia, and New Zealand.

The premature death rate was calculated as the number of deaths/the total population in the 30–69-year-old age group. The trends of premature death in the above countries were demonstrated to be basically consistent with our findings, i.e., early death due to AFF has constantly increased in recent decades. The detailed trends of early death in those countries are provided in Supplementary Figure S1.

#### 2.4.3. Age-standardized rate and estimated annual percentage change

The age-standardized rate (ASR) is a good indicator of disease and risk factor trends; therefore, we applied the widely used ASR-estimated annual percentage change (EAPC) to measure the magnitude of trend changes between 1990 and 2019, and 2020 and 2029 (22, 23). The ASR (per 100,000 individuals) was calculated based on the following formula:

$$\mathrm{ASR}=\frac{\sum_{i=1}^{A}a_{i}w_{i}}{\sum_{i=1}^{A}w_{i}}\times 100,000$$

where *a_i_* denotes the *i*th age class and the number of persons (or weight) (*w_i_*) in the same age subgroup *i* of the chosen reference standard population. The value was then divided by the sum of the standard population weights. It is assumed that the natural logarithm of the ASR is linear over time; thus, *Y *=* α + βX + ε*, where *Y* = ln (ASR), *X* = calendar year, and *ε* = the error term. The EAPC was calculated using the formula 100 × [exp(*β*) − 1], and the 95% CI was obtained from the linear regression model (24). The ASR was deemed to be increased if the EAPC and the lower boundary of its 95% CI were both >0 and decreased if the EAPC and the upper boundary of its 95% CI were both <0.

#### 2.4.4. Pearson's correlation coefficient

Pearson's correlation was used to analyze the relationship between the ratio of female to male mortality and SDI. Locally weighted scatter plot smoothing was used in the regression analysis to create a smooth line to visualize the relationship between variables. The greater the absolute value of R approaches 1, the stronger the correlation is, and a *p* value <0.05 was considered statistically significant.

#### 2.4.5. Percentage change and the contribution of different age groups to total death

The percentage change in the death rate between 1990 and 2019 was calculated as follows: [death rate in 2019 (or 2029) − death rate in 1990 (or 2019)]/death rate in 1990 (or 2019).

In addition, to reflect the age composition of the death burden, we first summed the number of deaths in all age groups (30–95+) in the same year and designated the value as the denominator (*T*); we then summed the death numbers of the 30–49-, 50–69-, and 70+-year-old groups and designated the values as the numerator (*t*); finally, the age composition was calculated using the formula *t*/*T* × 100%.

All statistics were performed using the R program (version 4.0.4; R Core Team). A *p* value <0.05 was considered statistically significant.

#### 2.4.6. Patient and public involvement

No additional patients or public were involved in this study, and no detailed information about patient or public involvement was disclosed by GBD due to its global-scale data collection and the public accessibility.

## 3. Results

### 3.1. Global AFF-related death burden between 1990 and 2019

**Figure 1:**
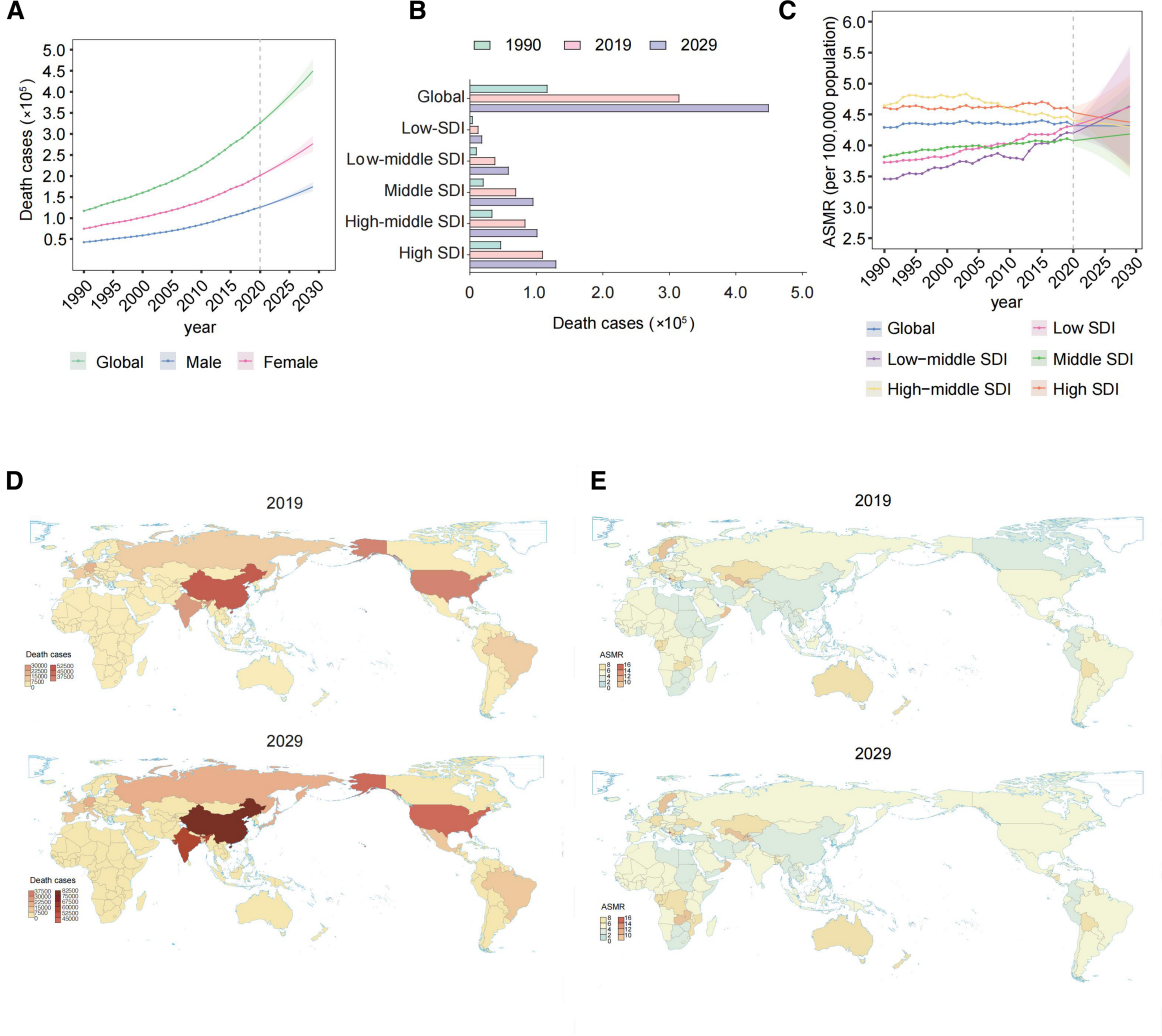
Global death cases and death rates of AFF from 1990 to 2019 and projection to 2029. (**A**) Deaths by sex from 1990 to 2019 and projection to 2029. (**B**) Deaths in territories with low to high SDIs in 1990, 2019, and 2029. (**C**) The ASMRs in different SDI regions from 1990 to 2019 and projection to 2029. (**D**) The death cases in 167 countries and territories in 2019 and 2029. (**E**) The ASMRs in 167 countries and territories in 2019 and 2029. AFF, atrial fibrillation and flutter; SDI, sociodemographic index; ASMR, age-standardized mortality rate; EAPC, estimated annual percentage change.

The global total number of AFF deaths increased progressively from 117,037.99 [95% uncertainty interval (UI) 103,695.27–138,452.31] in 1990 to 315,336.77 (95% UI 267,964.21–361,013.8) in 2019 (Table 1, Figure 1A). Moreover, the ASMR also gradually increased over the past three decades (4.29 per 100,000 individuals (95% UI 3.73–5.09) in 1990 and 4.38 (95% UI 3.7–5.05) in 2019), with an EAPC of 0.04 (95% CI 0.02–0.06). Both the number of deaths and ASMR in women distinctly surpassed those in men. More deaths were associated with AFF in the high-SDI region (Figure 1B). However, the ASMRs in high-middle-/high-SDI regions have started to decline in recent years, while low- to middle-SDI regions have experienced steady and significant increases (Figure 1C).

**Table 1:** The death cases and age-standardized death rates of atrial fibrillation and flutter at the level of global and five SDI regions from 1990 to 2029.

Characteristics	1990^a^		2019		2029		1990–2019^b^	2020–2029
	Number of deaths	ASMR per 100, 000	Number of deaths	ASMR per 100, 000	Number of deaths	ASMR per 100, 000	EAPC of ASMR	EAPC of ASMR
	No. (95% UI)	No. (95% UI)	No. (95% UI)	No. (95% UI)	No. (95% UI)	No. (95% UI)	No. (95% CI)	No. (95% CI)
Global								
Global	117,037.99 (103,695.27–138,452.31)	4.29 (3.73–5.09)	315,336.77 (267,964.21–361,013.80)	4.38 (3.70–5.05)	404,593.40 (376,960.16–432,226.65)	4.32 (3.65–4.99)	0.04 (0.02–0.06)	−0.01(−0.01 to 0.00)
Males	42,511.29 (33,946.40–54,885.12)	4.13 (3.23–5.37)	121,548.15 (97,352.46–148,260.66)	4.33 (3.46–5.33)	163,174.60 (151,941.90–174,407.29)	4.24 (3.55–4.92)	0.15 (0.13–0.17)	−0.06(−0.07 to −0.05)
Females	74,526.70 (64,121.28–89,412.20)	4.37 (3.70–5.26)	193,788.62 (160,729.04–224,866.70)	4.40 (3.65–5.11)	246,189.58 (227,905.76–264,473.40)	4.38 (3.68–5.09)	−0.01(−0.03 to 0.02)	0.052 (0.05–0.06)
SDI regions								
Low	4,502.23 (3,015.55–5,732.11)	3.73 (2.42–4.76)	13,159.62 (9,590.65–16,005.67)	4.30 (3.08–5.25)	19,964.65 (18,359.33–21,569.97)	4.62 (3.71–5.53)	0.50 (0.47–0.53)	0.76 (0.75–0.77)
Low-middle	10,542.53 (8,364.01–12,755.10)	3.46 (2.66–4.14)	38,423.23 (32,144.39–44,621.79)	4.21 (3.50–4.87)	60,986.69 (55,674.13–66,299.24)	4.64 (3.67–5.60)	0.61 (0.55–0.67)	1.11 (1.11–1.11)
Middle	20,777.69 (18,352.07–23,538.78)	3.82 (3.32–4.28)	69,793.44 (59,854.44–80,804.05)	4.11 (3.50–4.75)	101,188.81 (93,873.34–108,504.28)	4.18 (3.49–4.88)	0.21 (0.19–0.24)	0.29 (0.29–0.30)
High-middle	33,987.21 (30,154.52–43,292.18)	4.64 (4.04–5.92)	83,844.43 (70,922.60–100,863.46)	4.47 (3.78–5.39)	106,973.03 (97,560.64–116,385.41)	4.30 (3.50–5.10)	−0.25(−0.33 to −0.18)	−0.26(−0.28 to −0.24)
High	47,156.79 (40,195.42–59,598.44)	4.62 (3.93–5.82)	109,926.20 (86,253.87–132,025.38)	4.61 (3.67–5.52)	118,233.47 (108,505.54–127,961.40)	4.38(3.61–5.14)	0.03(0.01–0.06)	−0.39(−0.41 to −0.38)

ASMR, age-standardized mortality rate; EAPC, estimated annual percentage change; UI, uncertainty interval; CI, confidence interval.

^a^ Number of deaths and ASMR at the year of 1990.

^b^ EAPC of ASMR between the year of 1990 to 2019.

Regionally, 17 GBD regions had ASMRs that increased during the period (Table 2). At the national level, China, India, and the United States had the greatest number of deaths due to AFF in 2019 (all exceeding 20,000) (Figure 1D). Regarding the ASMR, the top ASMRs are in Montenegro, Qatar, and Bahrain (>10.00 per 100,000), while Singapore, Kuwait, and Japan have the lowest (<2.5 per 100,000) (Figure 1E).

**Table 2:** The death cases and age-standardized death rates of atrial fibrillation and flutter at the level of 21 GBD regions from 1990 to 2029.

Characteristics	1990^a^		2019		2029		1990–2019^b^	2020–2029
	Number of deaths	ASMR per 100, 000	Number of deaths	ASMR per 100, 000	Number of deaths	ASMR per 100, 000	EAPC of ASMR	EAPC of ASMR
	No. (95% UI)	No. (95% UI)	No. (95% UI)	No. (95% UI)	No. (95% UI)	No. (95% UI)	No. (95% CI)	No. (95% CI)
Eastern Sub–Saharan Africa	1,726.92 (1,013.28–2,265.27)	4.23 (2.37–5.58)	4,485.36 (2,872.26–5,572.06)	4.69 (2.98–5.87)	6,547.33 (5,905.89–7,188.76)	4.99 (3.80–6.19)	0.34 (0.25–0.44)	0.66 (0.63–0.69)
Western Sub–Saharan Africa	2,118.54 (1,665.21–2,779.08)	4.50 (3.55–5.93)	4,943.96 (4,096.87–5,798.52)	4.75 (3.89–5.59)	6,508.92 (5,851.80–7,166.05)	4.85 (3.76–5.94)	0.08 (0.03–0.12)	0.31 (0.30–0.32)
Oceania	61.07 (43.99–86.59)	4.10 (2.76–6.01)	171.04 (129.14–228.23)	4.25 (3.24–5.50)	232.65 (186.95–278.35)	4.37 (2.64–6.11)	0.17 (0.12–0.23)	0.32 (0.31–0.32)
Central Sub–Saharan Africa	562.79 (324.05–964.55)	5.00 (2.85–8.21)	1,650.88 (1,053.75–2,388.70)	5.63 (3.55–8.24)	2,458.90 (2,185.65–2,732.15)	6.05 (4.39–7.71)	0.37 (0.28–0.46)	0.80 (0.78–0.81)
South Asia	8,752.97 (6,601.77–11,314.43)	3.33 (2.41–4.27)	36,619.42 (28,651.51–45,925.96)	4.10 (3.18–5.15)	67,242.19 (58,148.30–76,336.08)	4.64 (3.14–6.13)	0.59 (0.46–0.71)	1.46 (1.45–1.47)
Central Latin America	2,588.04 (2,210.80–3,312.33)	4.46 (3.76–5.68)	9,907.14 (8,078.88–12,666.97)	4.56 (3.72–5.83)	14,142.59 (12,620.47–15,664.70)	4.94 (3.75–6.13)	−0.09(−0.17 to 0.00)	0.94 (0.93–0.95)
Caribbean	909.22 (768.20–1,067.87)	4.46 (3.72–5.28)	2,499.53 (2,064.22–3,049.12)	4.73 (3.91–5.79)	2,967.88 (2,598.32–3,337.44)	5.17 (3.75–6.59)	0.26 (0.16–0.37)	0.92 (0.92–0.92)
Andean Latin America	686.11 (572.11–782.01)	4.41 (3.67–5.06)	2,238.96 (1,803.70–2,716.92)	4.32 (3.48–5.24)	2,657.69 (2,307.88–3,007.50)	4.04 (2.89–5.20)	0.10 (0.02–0.19)	−0.65(−0.67 to −0.63)
Tropical Latin America	2,737.63 (2,337.69–3,421.06)	4.78 (3.99–5.95)	11,051.30 (8,832.39–13,042.57)	5.03 (4.01–5.94)	13,589.10 (12,000.56–15,177.64)	4.78 (3.47–6.08)	0.50 (0.31–0.70)	−0.48(−0.49 to −0.47)
Southern Sub–Saharan Africa	582.49 (490.64–654.33)	3.10 (2.59–3.50)	1,440.02 (1,257.71–1,587.49)	3.94 (3.38–4.37)	1,612.39 (1,365.19–1,859.58)	3.56 (2.32–4.81)	0.79 (0.58–1.00)	−1.01(−1.04 to −0.99)
Southeast Asia	4,641.66 (4,040.58–5,317.27)	3.19 (2.72–3.64)	16,028.17 (13,356.96–19,305.49)	3.99 (3.27–4.75)	22,152.57 (20,274.84–24,030.30)	4.10 (3.29–4.92)	0.76 (0.70–0.82)	0.39 (0.39–0.40)
North Africa and Middle East	3,462.66 (2,708.46–3,995.89)	3.48 (2.67–4.11)	10,504.69 (8,921.76–12,764.88)	3.66 (3.07–4.33)	13,611.32 (12,274.47–14,948.17)	3.70 (2.84–4.56)	0.19 (0.10–0.28)	0.20 (0.18–0.21)
East Asia	16,846.64 (14,177.55–19,679.32)	4.00 (3.31–4.63)	54,065.66 (45,776.98–62,320.18)	3.82 (3.19–4.42)	82,914.39 (75,426.71–90,402.08)	3.81 (3.02–4.61)	−0.26(−0.31 to −0.21)	0.08 (0.05–0.12)
Southern Latin America	1,782.67 (1,505.20–2,175.64)	4.92 (4.13–5.93)	4,639.46 (3,865.81–6,054.54)	5.32 (4.44–6.94)	5,071.25 (4,382.93–5,759.58)	5.48 (3.85–7.11)	0.29 (0.19–0.39)	0.29 (0.28–0.30)
Central Europe	5,140.77 (4,569.39–6,290.64)	4.51 (3.93–5.43)	10,882.03 (9,017.02–12,839.82)	4.83 (3.99–5.70)	13,128.10 (11,846.27–14,409.93)	4.94 (3.90–5.97)	0.15 (0.10–0.20)	0.31 (0.31–0.32)
Eastern Europe	8,495.61 (7,312.29–11,898.23)	4.03 (3.44–5.60)	15,981.79 (13,463.09–20,894.44)	4.62 (3.88–6.02)	23,619.43 (17,764.35–29,474.50)	4.71 (2.32–7.10)	0.22 (0.07–0.37)	0.33 (0.33–0.34)
Australasia	1,522.15 (1,251.28–1,741.82)	7.38 (5.96–8.41)	4,081.60 (3,258.25–4,976.87)	6.86 (5.45–8.33)	4,560.77 (3,998.25–5,123.29)	6.76 (5.08–8.43)	−0.44(−0.52 to −0.36)	0.00 (0.00–0.01)
Western Europe	33,269.38 (28,672.51–45,265.15)	5.77 (4.91–7.83)	69,927.95 (55,541.11–84,440.70)	5.76 (4.59–6.99)	72,846.44 (65,546.22–80,146.67)	5.34 (4.30–6.37)	0.06 (0.02–0.10)	−0.67(−0.69 to −0.65)
High–income North America	15,092.92 (12,600.08–18,698.80)	4.04 (3.36–4.99)	36,443.53 (29,294.80–44,398.90)	4.96 (4.01–6.05)	39,521.56 (36,323.20–42,719.92)	5.08 (4.12–6.04)	0.72 (0.63–0.80)	0.36 (0.35–0.38)
High–income Asia Pacific	4,773.88 (4,076.06–6,225.85)	3.00 (2.54–4.03)	15,307.29(11,653.49–20,614.67)	2.39(1.87–3.19)	17,593.15(15,434.26–19,752.05)	2.26(1.72–2.80)	−0.85(−0.95 to −0.75)	−0.47(−0.48 to −0.46)

ASMR, age-standardized mortality rate; EAPC, estimated annual percentage change; UI, uncertainty interval; CI, confidence interval.

^a^ Number of deaths and ASMR at the year of 1990.

^b^ EAPC of ASMR between the year of 1990 to 2019.

### 3.2. Greater mortality will shift from high-income regions to low-income regions in the next decade

Alarmingly, the AFF-related absolute death burden was projected to further increase. The number of deaths will reach 404,593.4 (95% UI 376,960.16–432,226.65) globally by 2029 (Figures 1A,B). Compared with that in 2019, the ASMR in 2029 will only slightly drop to 4.32 per 100,000 individuals (95% UI 3.65–4.99). Decreasing trends of death rates were also expected for both sexes, although the number of deaths will still increase. The pyramid structure of death cases from the low-SDI region (top) to the high-SDI region (bottom) was estimated to be the same as in 2019 (Figure 1B). And the continuing decreasing ASMRs in the high-middle-/high-SDI regions will remain (Figure 1C). However, the steady and significant ASMR growth is likely to lead low-/low-middle-SDI regions to exceed that in high-middle/high-SDI regions by 2029.

Regionally, 15 GBD regions were expected to experience ASMR growth and most of them were with relatively lower SDI (Table 1). At the national level, the countries with the highest and lowest numbers of death and ASMRs after 10 years were projected to be the same as in 2019 (Figures 1D,E).

### 3.3. Women have a higher ASMR than men globally, which is largely attributed to a disproportionately higher mortality rate in low-income regions

**Figure 2:**
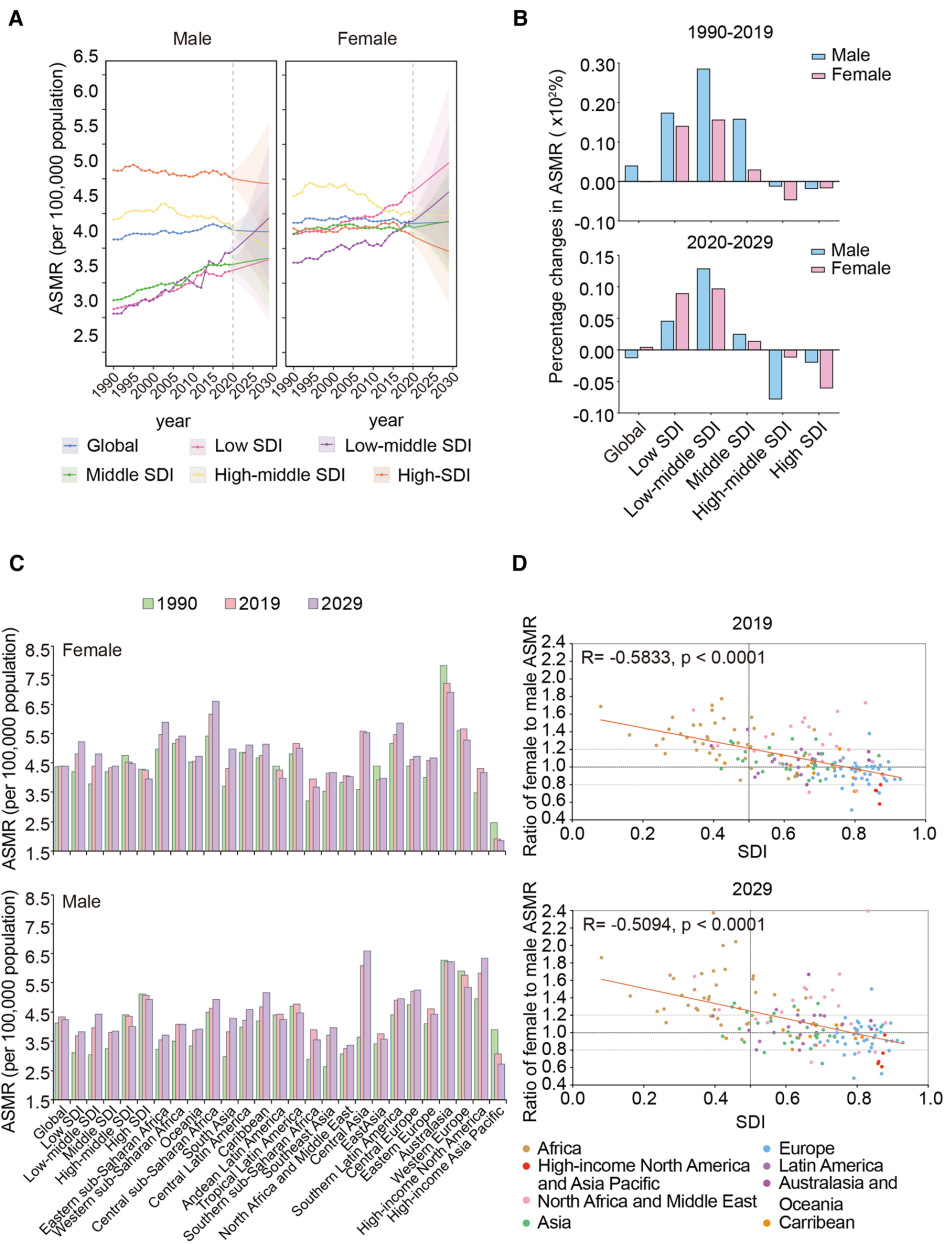
Sex differences in trends of AFF death rate in different regions from 1990 to 2019 and projection to 2029. (**A**) The ASMRs in different SDI regions by sex, from 1990 to 2019 and projection to 2029. (**B**) The percentage changes in female and male ASMRs in different SDI regions from 1990 to 2019 and projection to 2029. (**C**) The ASMRs globally, in territories with low to high SDIs, and in 21 GBD regions by sex in 1990, 2019, and 2029. (**D**) The correlation between the ratios of female to male ASMR in 167 countries and territories and their corresponding SDI levels in 2019 and 2029. AFF, atrial fibrillation and flutter; SDI, sociodemographic index; ASMR, age-standardized mortality rate.

The global ASMR of women was higher than that of men between 1990 and 2019 (Figure 2A). During this period, the female ASMR in low-, low-middle-, middle-, and middle-high-SDI regions were generally higher than those in men. However, the male ASMR was higher than that in women in the high-SDI region. In addition, the global female and male ASMRs were stable during the period; however, both significantly increased in low-/low-middle-SDI regions, especially for men, and the ASMRs surpassed that in high-middle/high-SDI regions recently. The largest percentage changes in ASMRs for the sexes occurred in the low-middle-SDI region, where men had higher increments than women (Figure 2B).

The highest female ASMR shifted from the high-middle-SDI region in 1990 to the low-SDI region in 2019 (Figure 2C). Australasia and high-income Asia Pacific were the two regions with the highest and lowest ASMRs of both sexes, respectively.

Interestingly, as the SDI increased, men gradually began overtaking women with respect to the ASMR (Figure 2D). The ratio of female to male ASMR in most African countries was >1.2; however, regarding Asia, Latin America, and Australasia, the differences were evenly distributed in the range of 0.8–1.2. When it comes to high-income North America and the Asia Pacific, the ratios were <0.8.

### 3.4. Women will maintain higher AFF-related mortality than men in the future

Between 2020 and 2029, the global female ASMR was predicted to be higher than the male ASMR (Figure 2A). Notably, by approximately 2025, the male ASMR in the low-middle-SDI region may exceed that in the high-middle-SDI region and have the second-highest male mortality due to AFF worldwide. In addition, the low-middle-SDI region will continue to experience a significant increase in ASMRs for both sexes (Figure 2B).

Regionally, Australasia remains to have the highest female ASMR in the future, despite its ASMRs decreasing yearly (Figure 2C). For men, the highest ASMR was predicted to shift from Australasia to Central Asia by 2029. Meanwhile, the lowest ASMRs for both sexes will still be in the high-income Asia Pacific.

Moreover, the intriguing phenomenon of the sex difference in ASMRs that we observed in different regions will hardly change by 2029 (Figure 2D). Notably, the sex differences in African countries may be further exacerbated (more countries will increase their ratios >1.6) and more European countries will change from one with a higher male ASMR to one with a higher female ASMR.

### 3.5. AFF-related premature mortality is continuously increasing worldwide

**Figure 3:**
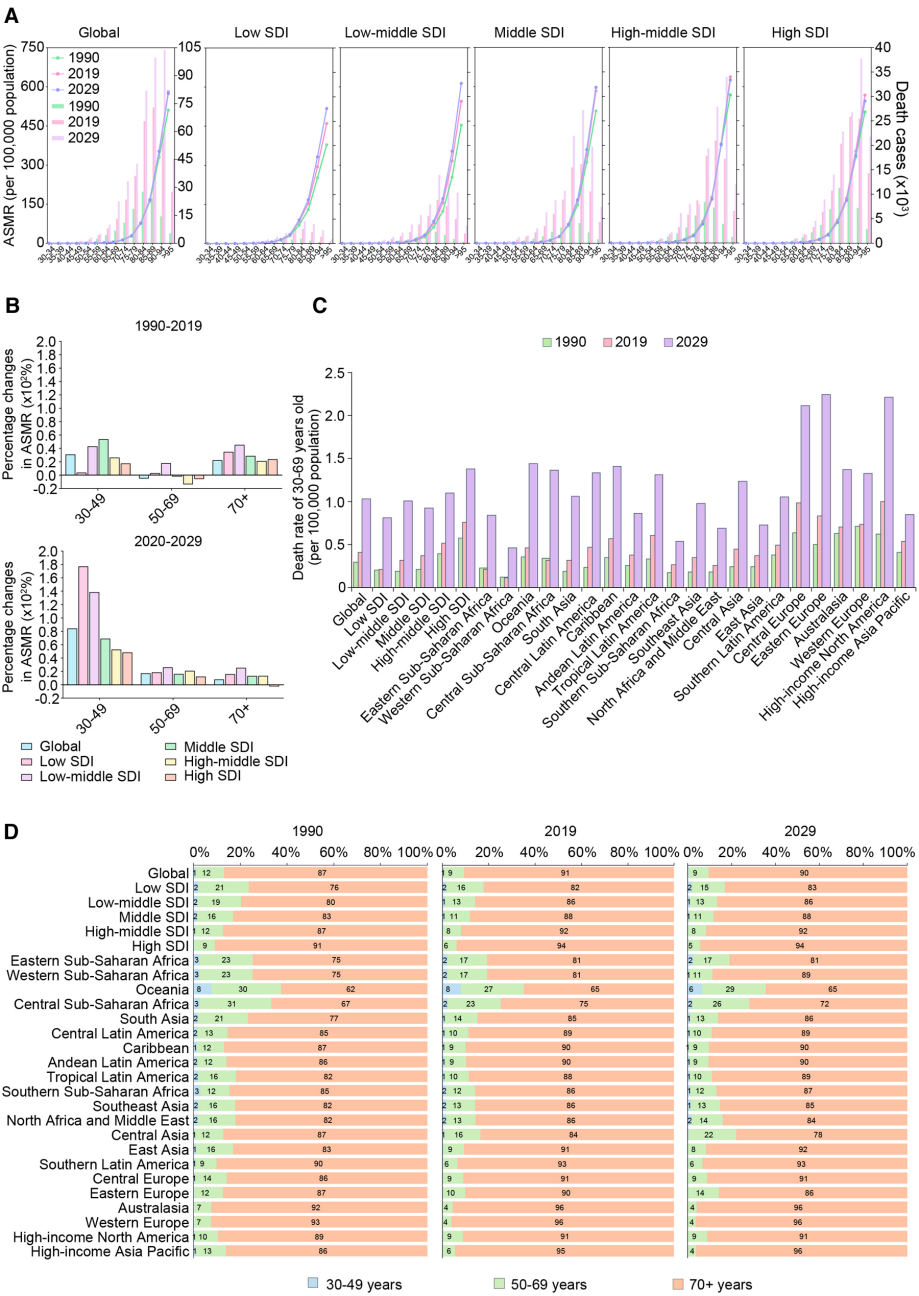
The trends of death cases and rates of AFF by age groups in different regions from 1990 to 2019 and projection to 2029. (**A**) The death cases and ASMRs in 30–95 plus years groups (5 years an interval) in 1990, 2019 and 2029 globally and in territories with low to high SDIs. (**B**) The percentage changes in ASMRs in three age groups (30–49 years, 50–69 years, and 70+ years) between 1990 and 2019 and 2019–2029 globally and in territories with low to high SDIs. (**C**) The premature ASMR (30–69 years old) globally, in territories with low to high SDIs and in 21 GBD regions in 1990, 2019, and 2029. (**D**) The age composition of (30–49, 50–69, and 70+ years old) globally, in territories with low to high SDIs and in 21 GBD regions in 1990, 2019, and 2029. AFF, atrial fibrillation and flutter; SDI, sociodemographic index; ASMR, age-standardized mortality rate; EAPC, estimated annual percentage change.

Globally and regionally, the ASMR increases with age. It was maintained at a very low level in the 30–69-year-old population but grew rapidly after the age of 70 years, reaching its peak among individuals aged 95+ years (Figure 3A). In addition, the peak number of deaths was basically concentrated in the 80+ age groups, but with the increasing SDI, which slid to the older population.

Notably, the ASMR in the 30–49-year-old group has increased particularly faster in low-middle- and middle-SDI regions (Figure 3B). After further analyzing the situation of premature death (at 30–69 years), we found that almost the whole world experienced an increase in early death between 1990 and 2019 (Figure 3C).

To further verify the trends of premature death, we analyzed the data from the WHO mortality database (Supplementary Figure S1). We found that in recent decades, premature death rates have constantly risen in the United States, the United Kingdom, Uzbekistan, Egypt, Australia, and other countries. These results are consistent with our findings.

The age compositions of the death cases showed that the proportion of elderly individuals increased with increasing SDI (Figure 3D). Oceania had the “youngest” distribution of deaths due to AFF while Western Europe had the “oldest.” Notably, Central Asia was the only region that experienced a declining age at death, as the older population accounted for 87% of AFF-related deaths in 1990 vs. 84% in 2019.

### 3.6. More regions will face an increased death burden in their young populations

Between 2020 and 2029, the ASMRs of the 75+-year age groups in low- to middle-SDI regions were expected to continue to grow (Figure 3A). Moreover, the peak death rates at the global and regional levels were all estimated to continuously move to older age groups over time.

Notably, the increase of ASMR in the 30–49-year-old group is conspicuous between 2019 and 2029, especially in low- and low-middle-SDI regions (Figure 3B). The growth of ASMR in the 30–49-year-old group was faster than those of the 50–69-year-old and 70+ age groups, indicating that early death may exacerbate in various regions in the future.

However, despite low-/low-middle-/middle-SDI regions having experienced relatively more rapid growth in every age group, the ASMR in people aged 30–69 years will still be higher in high-SDI regions in the future, and the highest ASMR was predicted to be in Eastern Europe by 2029 (Figure 3C). However, it is worth noting that more GBD regions with lower SDIs, including central sub-Saharan Africa, Southeast Asia, North Africa, the Middle East, and Central Asia, will experience a trend toward younger ages in AFF-related death (Figure 3D).

### 3.7. High systolic blood pressure/body mass index are the leading drivers of AFF death

**Figure 4:**
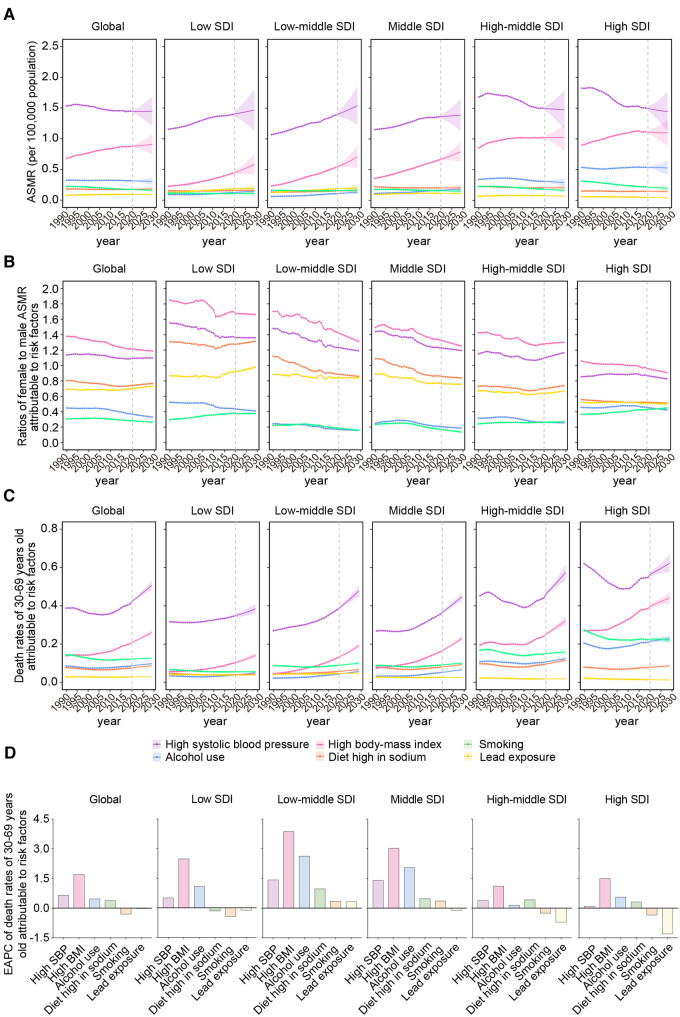
The predominant contributions of metabolic risk factors to AFF-related deaths by SDI, sex, and age groups. (**A**) The ASMRs attributable to main risk factors from 1990 to 2019 and projection to 2029. (**B**) The ratios of female to male ASMRs attributable to main risk factors from 1990 to 2019 and projection to 2029. (**C**) The ASMRs attributable to main risk factors in 30–69 years old population from 1990 to 2019 and projection to 2029 globally, in territories with low to high SDIs. (**D**) The EAPCs of ASMRs attributable to main risk factors in 30–69 years old population between 1990 and 2029 globally, in territories with low to high SDIs. AFF, atrial fibrillation and flutter; SDI, sociodemographic index; ASMR, age-standardized mortality rate; EAPC, estimated annual percentage change; SBP, systolic blood pressure; BMI, body mass index.

Between 1990 and 2019, high systolic blood pressure (SBP) and high body mass index (BMI) were the top two risk factors for AFF. In addition, globally, the ASMR attributed to high SBP maintained a high but steady decrease in recent decades, while high BMI was shown to keep growing over time (Figure 4A). Similar trends were observed in high-middle/high-SDI regions. However, both high SBP and high BMI in low-/low-middle-/middle-SDI regions had significantly increased.

High SBP and BMI were the top risk factors in all GBD regions (Supplementary Figure S2). As SDI increases, the ASMRs attributable to high SBP were gradually transformed from upward to solely decreasing. However, the changing pattern of high BMI was almost uniform in all the GBD regions to maintain stable and significant growth. These results were consistent with the findings at SDI levels.

The ratios of female ASMR to male ASMR attributable to the risk factors demonstrated that high SBP/BMI in women (>1.2) and alcohol use/smoking (<0.8) in men were the main resources of sex differences in AFF-related death (Figure 4B). These sex-specific discrepancies were more pronounced in regions with lower SDI. As the SDI increased, the male ASMR gradually began overtaking the female ASMR.

Notably, the global 30–69-year-old population was affected most by high SBP in the last 30 years, followed by high BMI, and both factors experienced a significant increase, especially in low- and low-middle-SDI regions (Figure 4C). Notably, the death contribution from high SBP had a substantial decline before 2010, but it had rebound later in high-middle- and high-SDI regions.

### 3.8. High BMI is the most rapidly rising contributor to premature death from AFF

High SBP and high BMI will remain the greatest contributors to AFF-associated death by 2029, Although the contribution from high SBP will be flattened at the global level, the contribution from high BMI was expected to be on the rising trend (Figure 4A). It is noteworthy that the contributions from high SBP are still rising rapidly from low- to middle-SDI regions. Due to the significant and unremitting increase, high SBP in the low-middle-SDI region may ultimately exceed that in high-income regions over the coming years. In the meantime, the rising trend of high BMI will level off in high-middle-/high-SDI regions, while the trends will rise more rapidly in the lower SDI regions.

Consistent with the results at the SDI level, the ASMRs attributable to high SBP in most GBD regions will be stable or decrease in the future; in certain low-income areas, such as Eastern sub-Saharan Africa, Central Latin America, and the Caribbean, these ASMRs are expected to continue increasing (Supplementary Figure S2). In sharp contrast, a high BMI may bear more of the death burden in the next decade in both low- or high-income regions.

Worryingly, the sex differences derived from the metabolic and behavioral risks in low-SDI regions will stabilize in the future, not like the other regions where the differences coming from high SBP/BMI were shown to keep a downward trend (Figure 4B), suggesting that the sex gap in low-SDI regions may further widen in the future.

Premature death attributable to high SBP/BMI was expected to keep growing worldwide during 2020–2029 (Figure 4C). High BMI was shown to be the most rapidly rising contributor to premature death in AFF, both globally and regionally (Figure 4D). In addition, the impacts of alcohol use and a diet high in sodium on people aged 30–69 years also increased, while smoking showed a downward trend globally and in most SDI regions. Notably, the low-middle- and middle-SDI regions owned the highest increments of attributable ASMRs in the majority of the risk factors. The accumulating risk factors in the younger generation will significantly increase the disease burden later in life.

## 4. Discussion

This study was the first to provide a comprehensive evaluation and projection of the global mortality from and risk factors for AFF between 1990 and 2029. The worldwide prevalence and incidence of AFF have all declined in recent decades (3). However, AFF-related ASMR is still increasing, reaching 4.38 per 100,000 in 2019. If the situation does not improve, the number of deaths will increase by another 30% to 0.40 million by 2029. Notably, greater AFF-related mortality is estimated to shift from high-income regions to low-income regions in the next 10 years. Moreover, AFF-related premature death is constantly increasing, especially in low-/low-middle-SDI regions. High BMI has become the most rapidly rising contributor to this burden globally and regionally.

The disease burden of AFF is still mostly considered a serious problem in high-income countries and is frequently overlooked in low-income countries. It is true that the incidence and prevalence of AFF in wealthy countries remain higher than those in other regions (25–28). However, drastic control of risk factors, advanced diagnostic, and novel therapeutic strategies also led to decreasing AFF-related mortality in these regions (3, 29). In comparison, with epidemic transition (30), a surge of cardiometabolic risk factors and limited healthcare access, the prevention and intervention of AFF is far from satisfactory in low-income countries (31, 32). Moreover, some low-income countries, such as China, have also begun to experience both a low reproductive rate and fast aging of the population (33). Therefore, the gap in AFF death burden between high- and low-income regions is continuously narrowing. Regions with lower SDI will eventually be responsible for a greater AFF death burden.

Globally, despite having a lowering prevalence and incidence of AFF (3), women have higher AFF-related mortality. However, such a paradoxical difference seems largely attributed to a disproportionately higher death rate in women than in men in low-income countries. For women in low-income regions, poorer access to sufficient medical care (32), more prevalent rheumatic heart disease (34) and obesity (35), and higher susceptibility of systolic hypertension-related AF (36) are all reasons leading to higher AFF-related mortality than that of men. Consequently, measures to decrease disparities in healthcare and risk prevention between the sexes should be taken immediately.

AFF-related death increases exponentially with age. However, AFF is now also greatly affecting young people. Mounting evidence has suggested that young adults (aged 18–45 years) have developed an increasingly unhealthy cardiovascular risk profile in recent decades (11). Meanwhile, alcohol abuse has become the leading cause of premature death in men aged 15–59 years (37). Furthermore, job strain resulting from working long hours or negative emotions have all proven to be able to trigger symptomatic AF (38, 39). Therefore, as the main labor force of society, young generations are facing both physical and mental risk-related death; if these risks cannot be effectively halted, more premature deaths can be foreseen.

Hypertension and obesity are the leading risk factors for AFF (11, 40) and they were predicted to remain this way in the next decade. Notably, although high SBP is currently the most significant contributor, the rapid increase in the contribution from high BMI should not be neglected. People born in the 1960s have more significant weight gain worldwide; between the 1980s and 2010s, obesity in children also substantially increased. Moreover, premature hypertension is increasingly considered to be majorly secondary to obesity (11). Therefore, obesity may soon develop as the top contributor to AFF-related mortality in young individuals. Especially for low-income regions, it is time to adopt population-wide health initiatives to prevent a greater AFF burden on young individuals as they age. A routine electrocardiogram examination every few years for young-middle-aged patients with a higher risk of AFF, making regulations on tobacco use, heavy alcohol consumption, and sugar-rich beverages, and ensuring standard working hours (35–40 h/week) may be of great help.

There are limitations to this study. First, the large differences in quality, comparability, accuracy, and missing degree of the data may lead to certain deviations in the estimations in the GBD study. Second, due to the lack of treatment and pathological data, we cannot calculate the proportions of valvular and non-valvular AFF in the study population. Likewise, paroxysmal, persistent, and permanent AFF and their progression also cannot be analyzed. Third, the data on other important cardiometabolic factors are also lacking, which further limits our analysis. Lastly, our analysis mainly focused on regional and national levels without further investigation of the impacts of local characteristics on the death rate of AFF, such as differences between urban and rural areas.

## 5. Conclusion

In summary, AFF will still be an important public health concern. The number of deaths from AFF will continue to grow over the next 10 years. Notably, greater AFF-related mortality rates were predicted to shift from high-income regions to low-income regions, primarily due to rapid increases in metabolic risk factors in lower SDI regions. In addition, women have a higher mortality rate than men globally, which is primarily attributed to a disproportionately higher death rate in women than men in low-income regions. Most importantly, premature death is constantly increasing; although high SBP currently has the highest contribution to AFF-related early death, the rapidly rising contribution from high BMI cannot be ignored among younger people.

## Data Availability

Publicly available datasets were analyzed in this study. These data can be found here: Global Burden of Disease (GBD): https://vizhub.healthdata.org/gbd-results/ (no accession number).
