# Supplementary material for: The rising death burden of atrial fibrillation and flutter in low-income regions and younger populations
Source: Front Epidemiol. 2023 Jun 5;3:1122790. doi: 10.3389/fepid.2023.1122790 (PMC10910937; doi:10.3389/fepid.2023.1122790)
Supplement: Supplementary file 1 [file Datasheet1.docx]

***Supplementary files***

**Supplementary Figure 1.** The trends of premature death in countries selected from World Health Organization mortality database.

**Supplementary Figure 2.** The trends of ASMRs attributable to main risk factors in GBD regions from 1990 to 2019 and projection to 2029.

**Supplementary Table 1.** 5 sociodemographic index (SDI) regions and their country compositions.

**Supplementary Table 2.** Checklist of accurate and transparent global health estimates reporting (ie. GATHER checklist).

**Supplementary Table 3.** Prediction accuracy validation of Bayesian age-period-cohort model (＜10% means acceptable).

**Supplementary text: detailed priors used in RW2 priors**

**Supplementary Table 1:** 5 sociodemographic index (SDI) regions and their country compositions.

| **SDI** **quintile** | **Countries** |
| --- | --- |
| **Low** | Somalia, Niger, Chad, Burkina Faso, Mali, Central African Republic, Burundi, Mozambique, Guinea, Afghanistan, Ethiopia, Sierra Leone, Benin, Guinea-Bissau, South Sudan, Liberia, Democratic Republic of the Congo, Malawi, Senegal, Papua New Guinea, Eritrea, Madagascar, Gambia, Uganda, Solomon Islands, Côte d'Ivoire, Yemen, Togo, Nepal, United Republic of Tanzania, Rwanda, Haiti, Pakistan. |
| **Low-middle** | Bhutan, Comoros, Djibouti, Cambodia, Angola, Zimbabwe, Bangladesh, Vanuatu, Cameroon, Lao People's Democratic Republic, Honduras, Mauritania, Sao Tome and Principe, Zambia, Lesotho, Kenya, Timor-Leste,Nigeria, Sudan, Nicaragua, Myanmar, Cabo Verde, Guatemala, Kiribati, Tajikistan, Marshall Islands, Morocco, Ghana, Democratic People's Republic of Korea, Maldives, Bolivia (Plurinational State of), India, Congo, El Salvador, Eswatini, Micronesia (Federated States of), Palestine, Tuvalu, Dominican Republic, Kyrgyzstan, Belize, Mongolia, Venezuela (Bolivarian Republic of). |
| **Middle** | Namibia, Viet Nam, Guyana, Nauru, Syrian Arab Republic, Philippines, Tokelau, Saint Vincent and the Grenadines, Uzbekistan, Colombia, Botswana, Suriname, Tonga, Paraguay, Brazil, Ecuador, Samoa, Peru, Mexico, Algeria, Gabon, Egypt, Indonesia, Fiji, Cuba, Grenada, Iran (Islamic Republic of), Saint Lucia, Turkmenistan, Iraq, Tunisia, South Africa, Costa Rica, Albania, Azerbaijan, Jamaica, Equatorial Guinea, China, Panama, Thailand, Armenia. |
| **Middle-high** | Sri Lanka, Republic of Moldova, Uruguay, Georgia, Mauritius, Argentina, ebanon, Libya, Niue, American Samoa, Bosnia and Herzegovina, Kazakhstan, Seychelles, Dominica, Jordan, Ukraine, Malaysia, Palau, Barbados, Antigua and Barbuda, Portugal, North Macedonia, Belarus, Saint Kitts and Nevis, Turkey, Bahrain, Trinidad and Tobago, Chile, Romania, Greenland, Bulgaria, Cook Islands, Serbia, Spain, Northern Mariana Islands, Oman, Hungary, Montenegro, Croatia, Greece, Bahamas, United States Virgin Islands, Italy, Malta, Poland, Israel, Russian Federation, Saudi Arabia. |
| **High** | Slovakia, Bermuda, Guam, Puerto Rico, Latvia, Brunei Darussalam, Czechia, Qatar, France, Estonia, Australia, New Zealand, Slovenia, Cyprus, Lithuania, United Kingdom, Austria, Belgium, Kuwait, Finland, United States of America, Singapore, Ireland, Taiwan (Province of China), Iceland, Japan, Sweden, Canada, Republic of Korea, United Arab Emirates, Netherlands, San Marino, Denmark, Andorra, Luxembourg, Germany, Monaco, Norway, Switzerland. |

**Supplementary Table 2**

**Checklist of accurate and transparent global health estimates reporting (ie. GATHER checklist).**

| **Item #** | **Checklist item** | **Reported on page #** |
| --- | --- | --- |
| **Objectives and funding** | | |
| **1** | Define the indicator(s), populations (including age, sex, and geographic entities), and time period(s) for which estimates were made. | Supplementary files page 2; Methods section. |
| **2** | List the funding sources for the work. | Page 10. Acknowledgements section. |
| **Data Inputs** | | |
| *For all data inputs from multiple sources that are synthesized as part of the study:* | | |
| **3** | Describe how the data were identified and how the data were accessed. | Supplementary files page 2; Methods/ study data. |
| **4** | Specify the inclusion and exclusion criteria. Identify all ad-hoc exclusions. | Supplementary files page 2; Methods/ study data. |
| **5** | Provide information on all included data sources and their main characteristics. For each data source used, report reference information or contact name/institution, population represented, data collection method, year(s) of data collection, sex and age range, diagnostic criteria or measurement method, and sample size, as relevant. | Supplementary files page 2; Methods/ study data. Available via online data source tools (<http://ghdx.healthdata.org/gbd-results-tool>) or https://www.who.int/data/data-collection-tools/who-mortality-database. |
| **6** | Identify and describe any categories of input data that have potentially important biases (e.g., based on characteristics listed in item 5). | Page 9. Discussion / “limitations” section. |
| *For data inputs that contribute to the analysis but were not synthesized as part of the study:* | | |
| **7** | Describe and give sources for any other data inputs. | Supplementary files page 2; Methods/ Study data. |
| *For all data inputs:* | | |
| **8** | Provide all data inputs in a file format from which data can be efficiently extracted (e.g., a spreadsheet rather than a PDF), including all relevant meta-data listed in item 5. For any data inputs that cannot be shared because of ethical or legal reasons, such as third-party ownership, provide a contact name or the name of the institution that retains the right to the data. | Supplementary files page 2; Methods/ Study data. Available via online data source tools (<http://ghdx.healthdata.org/gbd-results-tool>) or https://www.who.int/data/data-collection-tools/who-mortality-database. We do not have any data that cannot be shared. |
| **Data analysis** | | |
| **9** | Provide a conceptual overview of the data analysis method. A diagram may be helpful. | Supplementary files page 3-5: Methods/GBD estimation methods and Forecasting model development and validation section. |
| **10** | Provide a detailed description of all steps of the analysis, including mathematical formulae. This description should cover, as relevant, data cleaning, data pre-processing, data adjustments and weighting of data sources, and mathematical or statistical model(s). | Supplementary files page 4-7: Methods/Statistical analysis. |
| **11** | Describe how candidate models were evaluated and how the final model(s) were selected. | Supplementary files page 4-5. Methods/Forecasting model development and validation |
| **12** | Provide the results of an evaluation of model performance, if done, as well as the results of any relevant sensitivity analysis. | Supplementary files page 12: Table S2. |
| **13** | Describe methods for calculating uncertainty of the estimates. State which sources of uncertainty were, and were not, accounted for in the uncertainty analysis. | Supplementary files page 3 and 6: Methods section. |
| **14** | State how analytic or statistical source code used to generate estimates can be accessed. | Supplementary files page 2; Methods/ study data. |
| **Results and Discussion** | | |
| **15** | Provide published estimates in a file format from which data can be efficiently extracted. | No published estimates were used in our analysis. |
| **16** | Report a quantitative measure of the uncertainty of the estimates (e.g. uncertainty intervals). | Page 3-8. Results section. |
| **17** | Interpret results in light of existing evidence. If updating a previous set of estimates, describe the reasons for changes in estimates. | Page 8-9. Discussion section. |
| **18** | Discuss limitations of the estimates. Include a discussion of any modelling assumptions or data limitations that affect interpretation of the estimates. | Page 9. Discussion / “limitations” section. |

*This checklist should be used in conjunction with the GATHER statement and Explanation and Elaboration document, found on gather-statement.org*

**Supplementary Table 3**

**Prediction accuracy validation of Bayesian age-period-cohort model (＜10% means acceptable).**

| **Location** | **Average absolute percentage deviation** |
| --- | --- |
| Global | 1.474% |
| **Socio-demographic index** | 1.393% |
| Low | 1.567% |
| Low-middle | 4.256% |
| Middle | 0.990% |
| High-middle | 3.438% |
| High | 0.519% |
| **Region** | 1.393% |
| Eastern Sub-Saharan Africa | 0.438% |
| Western Sub-Saharan Africa | 0.694% |
| Oceania | 1.393% |
| Central Sub-Saharan Africa | 0.928% |
| South Asia | 3.054% |
| Central Latin America | 1.417% |
| Caribbean | 5.531% |
| Andean Latin America | 5.568% |
| Tropical Latin America | 1.190% |
| Southern Sub-Saharan Africa | 0.367% |
| Southeast Asia | 1.590% |
| North Africa and Middle East | 0.689% |
| Central Asia | 0.636% |
| East Asia | 2.624% |
| Southern Latin America | 2.609% |
| Central Europe | 3.268% |
| Eastern Europe | 3.161% |
| Australasia | 4.776% |
| Western Europe | 0.589% |
| High-income North America | 0.729% |
| High-income Asia Pacific | 3.033% |

**Supplementary text: detailed priors used in RW2 priors**

Due to the expectation that effects adjacent in time might be similar, smoothing priors are commonly used for age, period, and cohort effects^1 2^. A standard choice is the second-order random walk (RW2)^2 3^, which assumes independent mean-zero normal distributions (with unknown variance) on the second differences of all time effects. This is a natural target for smoothing, as the second differences in APC models are identifiable^4^.

Consider the age effects, say, then the RW2 prior is given by：

$$f(\theta|\kappa_{\theta})\propto{\kappa_{\theta}}^{\frac{a-2}{2}}\exp\left( {-\frac{\kappa_{\theta}}{2}\sum_{a=3}^{A} (\theta_{a}-2\theta_{a-1}+\theta_{a-2})}^{2} \right)={\kappa_{\theta}}^{\frac{A-2}{2}}exp(-\frac{1}{2}\theta^{T}Q\theta)$$

$$Q=\kappa_{\theta}\left( \begin{matrix} 1 & -2 & 1 & & & & \\ -2 & 5 & -4 & 1 & & & \\ 1 & -4 & 6 & -4 & 1 & & \\ & & & & & & \\ & \ddots& \ddots& \ddots& \ddots& \ddots& \\ & & & & & & \\ & & 1 & -4 & 6 & -4 & 1 \\ & & & 1 & -4 & 5 & -2 \\ & & & & 1 & -2 & 1 \end{matrix} \right)$$

where$\kappa_{\theta}^{-1}$ denotes the variance parameter. Note that $Q$ is not of full rank so the RW2 is an example of an intrinsic Gaussian Markov field^3^. The RW2 penalizes deviations from a linear trend and is regarded as the discrete-time analogue of a cubic smoothing spline^5^. Alternatively, a linear drift component^4^ $z_{at}\sim N(0,{\kappa_{z}}^{-1})$ can be specified, which can be seen as a limiting case of the RW2 as the random walk variance goes to zero. To adjust for overdispersion additional independent mean-zero Gaussian random effects can be added to the linear predictor log($\frac{p_{at}}{1-p_{at}}$)^1 2^.

Depending on the model formulation, that is, the number of RW2 components and the inclusion of an overdispersion component, there are up to four unknown variance parameters. For computational convenience often inverse gamma distributions are assumed for these parameters, where the shape and rate parameter are appropriately defined.

**Reference**

1. Knorr-Held L, Rainer E. Projections of lung cancer mortality in West Germany: a case study in Bayesian prediction. *Biostatistics* 2001;2(1):109-29. doi: 10.1093/biostatistics/2.1.109 [published Online First: 2003/08/23]

2. Besag J, Green P, Mengersen HK. [Bayesian Computation and Stochastic Systems]: Rejoinder. *Statistical Science* 1995;10(1995)

3. Rue H, Held L. Gaussian Markov Random Fields. *Chapman & Hall/crc Boca* 2005;49(3):xii,263.

4. Clayton D, Schifflers E. Models for temporal variation in cancer rates. II: Age-period-cohort models. *Stat Med* 1987;6(4):469-81. doi: 10.1002/sim.4780060406 [published Online First: 1987/06/01]

5. Fahrmeir L, Tutz G. Multivariate Statistical Modelling Based on Generalized Linear Models. *Journal of the American Statistical Association* 1996;91(434)
