# Supplementary material for: The rising death burden of atrial fibrillation and flutter in low-income regions and younger populations
Source: Front Epidemiol. 2023 Jun 5;3:1122790. doi: 10.3389/fepid.2023.1122790 (PMC10910937; doi:10.3389/fepid.2023.1122790)
Supplement: Supplementary file 2 [file Image1.pdf]

Supplementary Figure 1

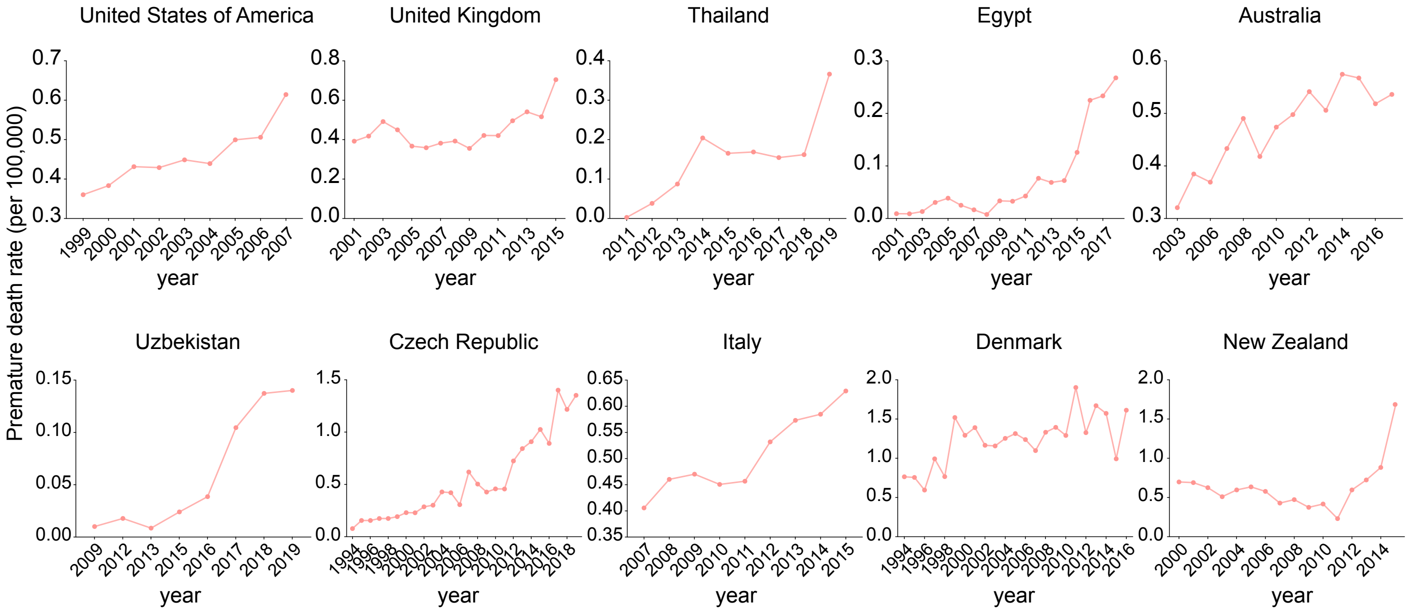

Supplementary Figure 1. The trends of premature death in countries selected from World Health Organization mortality database.
