# Supplementary material for: The rising death burden of atrial fibrillation and flutter in low-income regions and younger populations
Source: Front Epidemiol. 2023 Jun 5;3:1122790. doi: 10.3389/fepid.2023.1122790 (PMC10910937; doi:10.3389/fepid.2023.1122790)
Supplement: Supplementary file 3 [file Image2.pdf]

Supplementary Figure 2

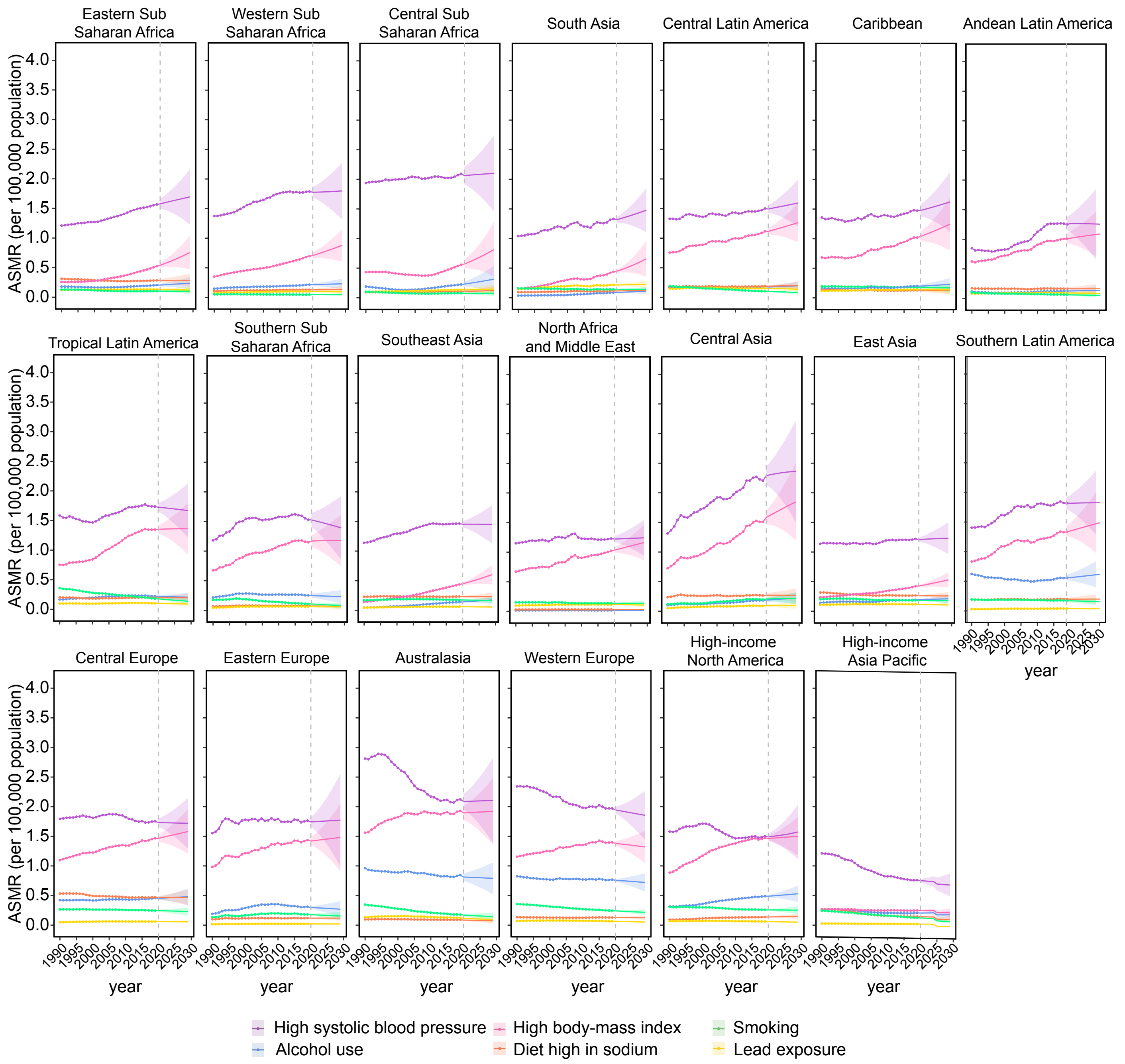

Supplementary Figure 2. The trends of ASMRs attributable to main risk factors in GBD regions from 1990 to 2019 and projection to 2029.
